# Supplementary material for: Major intensification of Atlantic overturning circulation at the onset of Paleogene greenhouse warmth
Source: Nat Commun. 2018 Nov 23;9:4954. doi: 10.1038/s41467-018-07457-7 (PMC6251870; doi:10.1038/s41467-018-07457-7)
Supplement: Supplementary file 1 — Supplementary Information [file 41467_2018_7457_MOESM1_ESM.pdf]

**Major intensification of Atlantic overturning circulation at the onset of Paleogene greenhouse warmth**

S. J. BATENBURG, S. VOIGT, O. FRIEDRICH, A.H. OSBORNE, A. BORNEMANN, T. KLEIN, L. PÉREZ-DÍAZ, M. FRANK

## Supplementary information

## Supplementary Tables

**Supplementary Table 1.** Overview of ocean drilling sites used in this study

| program | Exp. | Site/ Hole | Ocean | Region | Latitude, Longitude | Water depth (m) | Paleo depth (m) | Age model[a] | Notes |
|---|---|---|---|---|---|---|---|---|---|
| DSDP | 72 | 516F | South Atlantic | Rio Grande Rise | 30°16.59'S 35°17.10'W | 2109[1] | 500–1000[1] | Shipboard[1]; Magnetostratigraphy[2,3] | |
| DSDP | 74 | 525A | South Atlantic | Walvis Ridge | 29°04.24'S 02°59.12'E | 2467[4] | ~1300[4, 5] | Shipboard[4]; Magnetostratigraphy[6,3] | $\varepsilon_{Nd}$ data 69.2–72.0 Ma[7] |
| ODP | 208 | 1267 | South Atlantic | Walvis Ridge | 28°5.88'S 1°42.66'E | 4355[8] | ~3000[8] | Precession cycles[9]; Magnetostratigraphy[8,3] | Precession for Paleocene only[9] |
| DSDP | 41 | 369 | North Atlantic | Canary Islands | 26°35.5'N 14°59. 'W | 1752[10] | 1500–1700[8] | Shipboard[10] | |
| IODP | 342 | U1403 | North Atlantic | Newfound-land | 39°56.5997'N 51°48.1998'W | 4955.7[11] | 4000–4500[11] | Calcareous nannofossils[12] Cyclostratigraphy[13]; C-isotope stratigraphy[13] | Coordinates, depth: Hole A |

[a)] The tie-points used for establishing age models are listed in Supplementary Table 7.

**Supplementary Table 2.** Nd isotope results for Site 516F

| Sample ID[a] | Lab[b] | Site | Hole | Core | Sec. | from (cm) | to (cm) | Depth (mcd) | Age (Ma)[c] | $^{143}Nd/^{144}Nd$ | $\varepsilon_{Nd(0)}$[d] | $\boldsymbol{\varepsilon_{Nd(t)}}$[e] | 2σ[f] |
|---|---|---|---|---|---|---|---|---|---|---|---|---|---|
| *leachates* | | | | | | | | | | | | | |
| 14 | K | 516 | F | 83 | 3 | 130 | 132 | 904.91 | 56.43 | 0.5121558 | -9.41 | **-8.88** | 0.15 |
| 09 | K | 516 | F | 84 | 3 | 90 | 92 | 914.01 | 57.24 | 0.5121517 | -9.49 | **-8.96** | 0.07 |
| 11 | K | 516 | F | 85 | 2 | 21 | 23 | 921.32 | 58.74 | 0.5121629 | -9.27 | **-8.72** | 0.15 |
| 04 | K | 516 | F | 86 | 4 | 34 | 35 | 933.95 | 59.34 | 0.5121923 | -8.69 | **-8.15** | 0.07 |
| 05 | K | 516 | F | 87 | 4 | 59 | 61 | 943.7 | 61.76 | 0.5120942 | -10.61 | **-10.04** | 0.15 |
| 24 | O | 516 | F | 88 | 2 | 92 | 94 | 950.53 | 63.68 | 0.5120914 | -10.66 | **-10.07** | 0.13 |
| 13 | K | 516 | F | 89 | 3 | 27 | 29 | 960.88 | 64.69 | 0.5120655 | -11.17 | **-10.57** | 0.15 |
| 22 | O | 516 | F | 90 | 3 | 44 | 46 | 970.55 | 66.35 | 0.5120697 | -11.09 | **-10.47** | 0.13 |
| 10 | K | 516 | F | 91 | 3 | 120 | 122 | 980.81 | 67.08 | 0.5121035 | -10.43 | **-9.81** | 0.15 |
| 08 | K | 516 | F | 92 | 2 | 20 | 22 | 987.81 | 67.58 | 0.5121052 | -10.39 | **-9.77** | 0.15 |
| 08b | O | 516 | F | 92 | 2 | 20 | 22 | 987.81 | 67.51 | 0.5121159 | -10.18 | **-9.56** | 0.20 |
| 20 | O | 516 | F | 93 | 3 | 122 | 124 | 999.83 | 68.45 | 0.5121132 | -10.24 | **-9.60** | 0.13 |
| 20b | O | 516 | F | 93 | 3 | 122 | 124 | 999.83 | 68.45 | 0.5121221 | -10.06 | **-9.43** | 0.12 |
| 03 | K | 516 | F | 94 | 3 | 126 | 128 | 1009.37 | 69.14 | 0.5121107 | -10.29 | **-9.65** | 0.15 |
| 15 | K | 516 | F | 95 | 4 | 18 | 20 | 1019.29 | 70.12 | 0.5121276 | -9.96 | **-9.31** | 0.15 |
| 15b | O | 516 | F | 95 | 4 | 18 | 20 | 1019.29 | 70.07 | 0.5121394 | -9.73 | **-9.08** | 0.20 |
| 28 | O | 516 | F | 96 | 2 | 28 | 30 | 1025.39 | 70.75 | 0.5121296 | -9.92 | **-9.26** | 0.13 |
| 17 | K | 516 | F | 97 | 2 | 92 | 94 | 1032.03 | 71.45 | 0.5121532 | -9.46 | **-8.80** | 0.15 |
| 17b | O | 516 | F | 97 | 2 | 92 | 94 | 1032.03 | 71.40 | 0.5121423 | -9.67 | **-9.01** | 0.20 |
| 07 | K | 516 | F | 98 | 3 | 110 | 112 | 1036.71 | 71.78 | 0.5121286 | -9.94 | **-9.27** | 0.15 |
| 18 | K | 516 | F | 99 | 1 | 29 | 31 | 1041.9 | 72.07 | 0.5121324 | -9.86 | **-9.20** | 0.15 |
| 12 | K | 516 | F | 100 | 2 | 110 | 112 | 1046.71 | 72.50 | 0.5121225 | -10.06 | **-9.39** | 0.15 |
| *Detrital fractions*[g] | | | | | | | | | | | | | |
| 335 | O | 516 | F | 84 | 3 | 74 | 75 | 914.10 | 57.26 | 0.5121320 | -9.87 | **-9.35** | 0.31 |
| | | | | | 3 | 124 | 125 | | | | | | |
| 336 | O | 516 | F | 86 | 4 | 24 | 25 | 934.10 | 59.35 | 0.5122149 | -8.25 | **-7.71** | 0.23 |
| | | | | | 4 | 74 | 75 | | | | | | |
| 333 | O | 516 | F | 88 | 2 | 25 | 26 | 950.11 | 63.64 | 0.5120742 | -11.00 | **-10.42** | 0.39 |
| | | | | | 2 | 75 | 76 | | | | | | |
| 334 | O | 516 | F | 92 | 1 | 128 | 129 | 987.63 | 67.57 | 0.5120367 | -11.73 | **-11.13** | 0.23 |
| | | | | | 2 | 25 | 26 | | | | | | |
| 332 | O | 516 | F | 96 | 2 | 25 | 26 | 1025.61 | 70.78 | 0.5120939 | -10.61 | **-9.98** | 0.23 |
| | | | | | 2 | 75 | 76 | | | | | | |

[a)] Occurrences of "b" behind sample numbers indicate replicate measurements, averages are plotted.
[b)] The "K" stands for the geochemistry facilities of the GEOMAR in Kiel, Germany; "O" stands for the Department of Earth Sciences in Oxford, UK.
[c)] The tie-points used for establishing age models are listed in Supplementary Table 6.
[d)] Calculated as εNd(0)=
[e)] Calculated as εNd(t)= , using a Sm/Nd ratio of 0.124 (ref. 19) and a current value of CHUR of 0.512638 (ref. 34)
[f)] 2σ based on Jndi, expressed as $\varepsilon_{Nd}$.
[g)] each sample ID represent two samples that were combined to obtain sufficient material.

**Supplementary Table 3.** Nd isotope results for Hole 525A

| Sample ID[a] | Lab[b] | Site | Hole | Core | Sec. | from (cm) | to (cm) | Depth (mbsf) | Age (Ma)[c] | $^{143}Nd/^{144}Nd$ | $\varepsilon_{Nd(0)}$[d] | $\boldsymbol{\varepsilon_{Nd(t)}}$[e] | $2\sigma$[f] |
|---|---|---|---|---|---|---|---|---|---|---|---|---|---|
| *leachates* | | | | | | | | | | | | | |
| 13 | K | 525 | A | 31 | 1 | 89 | 91 | 375.00 | 55.99 | 0.5122055 | -8.44 | **-7.92** | 0.31 |
| 14 | K | 525 | A | 31 | 4 | 40 | 42 | 379.01 | 56.13 | 0.5121828 | -8.88 | **-8.36** | 0.31 |
| 12 | K | 525 | A | 32 | 2 | 40 | 42 | 385.51 | 56.36 | 0.5121794 | -8.95 | **-8.42** | 0.31 |
| 1 | K | 525 | A | 32 | 7 | 51 | 53 | 393.12 | 56.63 | 0.5121760 | -9.01 | **-8.49** | 0.31 |
| 5 | K | 525 | A | 35 | 2 | 70 | 72 | 404.81 | 57.05 | 0.5121955 | -8.63 | **-8.10** | 0.41 |
| 4 | K | 525 | A | 35 | 4 | 129 | 131 | 408.40 | 57.17 | 0.5122014 | -8.52 | **-7.99** | 0.41 |
| 2 | K | 525 | A | 37 | 1 | 36 | 38 | 421.97 | 59.41 | 0.5122931 | -6.73 | **-6.18** | 0.54 |
| 8 | K | 525 | A | 38 | 3 | 58 | 60 | 434.69 | 61.82 | 0.5122163 | -8.23 | **-7.65** | 0.26 |
| 11 | K | 525 | A | 39 | 2 | 47 | 49 | 442.58 | 63.32 | 0.5121830 | -8.88 | **-8.29** | 0.31 |
| 10 | K | 525 | A | 39 | 5 | 46 | 48 | 447.07 | 64.88 | 0.5123288 | -6.03 | **-5.43** | 0.31 |
| 22 | K | 525 | A | 40 | 2 | 71 | 73 | 452.32 | 66.08 | 0.5122363 | -7.84 | **-7.22** | 0.54 |
| 6 | K | 525 | A | 40 | 4 | 36 | 38 | 454.97 | 66.28 | 0.5122807 | -6.97 | **-6.36** | 0.41 |
| 7 | K | 525 | A | 41 | 1 | 15 | 17 | 459.76 | 66.70 | 0.5123508 | -5.60 | **-4.98** | 0.41 |
| 23 | K | 525 | A | 41 | 4 | 68 | 70 | 464.79 | 67.19 | 0.5123205 | -6.19 | **-5.57** | 0.54 |
| 3 | K | 525 | A | 42 | 1 | 109 | 111 | 470.20 | 67.72 | 0.5123062 | -6.47 | **-5.85** | 0.41 |
| 20 | K | 525 | A | 42 | 4 | 80 | 82 | 474.41 | 68.13 | 0.5123252 | -6.10 | **-5.47** | 0.54 |
| 24 | K | 525 | A | 43 | 1 | 41 | 43 | 479.02 | 68.55 | 0.5123169 | -6.26 | **-5.63** | 0.41 |
| 19 | K | 525 | A | 43 | 4 | 55 | 57 | 483.66 | 68.87 | 0.5122948 | -6.69 | **-6.06** | 0.54 |

[a–f] Footnotes as for Table 2.

**Supplementary Table 4.** Nd isotope results for Site 1267

| Sample ID[a] | Lab[b] | Site | Hole | Core | Sec. | from (cm) | to (cm) | Depth (CCSF-A) | Age (Ma)[c] | $^{143}Nd/^{144}Nd$ | $\varepsilon_{Nd(0)}$[d] | $\boldsymbol{\varepsilon_{Nd(t)}}$[e] | 2σ[f] |
|---|---|---|---|---|---|---|---|---|---|---|---|---|---|
| *leachates* | | | | | | | | | | | | | |
| 72 | K | 1267 | B | 23 | 4 | 15 | 17 | 232.41 | 56.08 | 0.5121009 | -10.48 | **-9.96** | 0.42 |
| 94 | K | 1267 | A | 23 | 3 | 85 | 87 | 237.36 | 56.42 | 0.5122368 | -7.83 | **-7.30** | 0.42 |
| 89 | K | 1267 | B | 24 | 2 | 88 | 90 | 242.34 | 56.69 | 0.5121505 | -9.51 | **-8.99** | 0.26 |
| 90 | K | 1267 | B | 24 | 5 | 131 | 133 | 247.27 | 57.01 | 0.5121974 | -8.59 | **-8.07** | 0.26 |
| 75 | K | 1267 | B | 25 | 1 | 43 | 45 | 252.25 | 57.34 | 0.5121350 | -9.81 | **-9.28** | 0.42 |
| 77 | K | 1267 | B | 25 | 4 | 89 | 91 | 257.21 | 57.64 | 0.5121368 | -9.78 | **-9.24** | 0.42 |
| 91 | K | 1267 | A | 25 | 4 | 6 | 8 | 262.17 | 57.94 | 0.5122271 | -8.01 | **-7.48** | 0.26 |
| 70 | K | 1267 | A | 26 | 1 | 36 | 38 | 267.07 | 58.23 | 0.5122038 | -8.47 | **-7.93** | 0.41 |
| 71 | K | 1267 | A | 26 | 4 | 86 | 88 | 272.07 | 58.66 | 0.5121706 | -9.12 | **-8.57** | 0.42 |
| 84 | K | 1267 | B | 27 | 5 | 139 | 141 | 277.05 | 59.19 | 0.5122532 | -7.51 | **-6.96** | 0.26 |
| 121 | K | 1267 | B | 28 | 2 | 141 | 143 | 282.02 | 59.85 | 0.5122049 | -8.45 | **-7.89** | 0.42 |
| 88 | K | 1267 | B | 28 | 6 | 37 | 39 | 286.98 | 60.51 | 0.5121909 | -8.72 | **-8.16** | 0.26 |
| 81 | K | 1267 | B | 29 | 3 | 47 | 49 | 291.93 | 61.16 | 0.5122020 | -8.51 | **-7.94** | 0.42 |
| 82 | K | 1267 | A | 29 | 1 | 20 | 22 | 296.91 | 61.82 | 0.5121558 | -9.41 | **-8.83** | 0.26 |
| 87 | K | 1267 | A | 29 | 4 | 66 | 68 | 301.87 | 62.56 | 0.5121470 | -9.58 | **-9.00** | 0.26 |
| 85 | K | 1267 | B | 30 | 7 | 43 | 45 | 306.84 | 63.45 | 0.5121989 | -8.57 | **-7.98** | 0.26 |
| 74 | K | 1267 | A | 30 | 4 | 70 | 72 | 311.76 | 64.50 | 0.5121065 | -10.37 | **-9.77** | 0.41 |
| 69 | K | 1267 | B | 32 | 1 | 125 | 127 | 316.61 | 65.46 | 0.5121669 | -9.19 | **-8.58** | 0.41 |
| 95 | K | 1267 | B | 32 | 3 | 48 | 50 | 318.84 | 65.79 | 0.5121637 | -9.25 | **-8.64** | 0.26 |
| 80 | K | 1267 | B | 32 | 5 | 65 | 67 | 321.61 | 66.04 | 0.5121628 | -9.27 | **-8.66** | 0.26 |
| 79 | K | 1267 | A | 31 | 7 | 121 | 123 | 326.65 | 66.38 | 0.5121709 | -9.11 | **-8.50** | 0.26 |
| 96 | K | 1267 | B | 33 | 4 | 104 | 106 | 331.55 | 66.72 | 0.5122319 | -7.92 | **-7.30** | 0.26 |
| 93 | K | 1267 | B | 34 | 1 | 76 | 78 | 336.57 | 67.06 | 0.5122705 | -7.17 | **-6.55** | 0.26 |
| 73 | K | 1267 | B | 34 | 4 | 122 | 124 | 341.53 | 67.39 | 0.5121906 | -8.73 | **-8.10** | 0.41 |
| 86 | K | 1267 | B | 35 | 1 | 70 | 72 | 346.66 | 67.74 | 0.5121759 | -9.01 | **-8.39** | 0.26 |
| 92 | K | 1267 | B | 35 | 4 | 128 | 130 | 351.74 | 68.09 | 0.5122110 | -8.33 | **-7.70** | 0.26 |
| 83 | K | 1267 | B | 36 | 1 | 2 | 4 | 356.93 | 68.44 | 0.5121910 | -8.72 | **-8.09** | 0.26 |
| 78 | K | 1267 | B | 36 | 4 | 48 | 50 | 361.89 | 68.78 | 0.5122140 | -8.27 | **-7.63** | 0.41 |
| 76 | K | 1267 | B | 36 | 7 | 34 | 36 | 366.25 | 69.07 | 0.5122616 | -7.34 | **-6.70** | 0.41 |
| *detrital fractions* | | | | | | | | | | | | | |
| 131TD | K | 1267 | A | 23 | 3 | 85 | 87 | 237.36 | 56.42 | 0.5121827 | -8.88 | **-8.36** | 0.18 |
| 129TD | K | 1267 | A | 25 | 4 | 6 | 8 | 262.17 | 57.94 | 0.5122021 | -8.50 | **-7.97** | 0.18 |
| 128TD | K | 1267 | B | 28 | 6 | 37 | 39 | 286.98 | 60.51 | 0.5122091 | -8.37 | **-7.81** | 0.18 |
| 126TD | K | 1267 | A | 30 | 4 | 70 | 72 | 311.76 | 64.50 | 0.5122047 | -8.45 | **-7.86** | 0.18 |
| 130TD | K | 1267 | B | 34 | 1 | 76 | 78 | 336.57 | 67.06 | 0.5120998 | -10.50 | **-9.88** | 0.18 |
| 127TD | K | 1267 | B | 36 | 4 | 48 | 50 | 361.89 | 68.78 | 0.5120398 | -11.67 | **-11.03** | 0.18 |

[a–f] Footnotes as for Table 2.

**Supplementary Table 5.** Nd isotope results for Site 369

| Sample ID[a] | Lab[b] | Site | Hole | Core | Sec. | from (cm) | to (cm) | Depth (CCSF-A) | Age (Ma)[c] | $^{143}Nd/^{144}Nd$ | $\varepsilon_{Nd(0)}$[d] | $\boldsymbol{\varepsilon_{Nd(t)}}$[e] | $2\sigma$[f] |
|---|---|---|---|---|---|---|---|---|---|---|---|---|---|
| *leachates* | | | | | | | | | | | | | |
| 98 | K | 369 | A | 36 | 1 | 49 | 51 | 374.99 | 68.39 | 0.5119294 | -13.82 | **-13.19** | 0.42 |
| 100 | K | 369 | A | 36 | 3 | 101 | 103 | 378.51 | 70.13 | 0.5119718 | -13.00 | **-12.35** | 0.42 |
| 103 | K | 369 | A | 36 | 5 | 119 | 121 | 381.69 | 71.64 | 0.5120641 | -11.19 | **-10.53** | 0.42 |
| 101 | K | 369 | A | 37 | 1 | 97 | 99 | 384.97 | 72.28 | 0.5119107 | -14.19 | **-13.52** | 0.26 |
| 101b | O | 369 | A | 37 | 1 | 97 | 99 | 384.97 | 72.28 | 0.5119545 | -13.33 | **-12.67** | 0.20 |
| 105 | O | 369 | A | 37 | 3 | 78 | 80 | 387.78 | 72.83 | 0.5118726 | -14.93 | **-14.26** | 0.31 |
| 105b | O | 369 | A | 37 | 3 | 78 | 80 | 387.78 | 72.83 | 0.5118352 | -15.66 | **-14.99** | 0.20 |
| *detrital fractions* | | | | | | | | | | | | | |
| 132TD | K | 369 | A | 36 | 3 | 101 | 103 | 378.51 | 70.13 | 0.5118415 | -15.54 | **-14.89** | 0.18 |
| 134TD | K | 369 | A | 37 | 3 | 78 | 80 | 387.78 | 72.83 | 0.5117438 | -17.44 | **-16.77** | 0.18 |
| 134TDb | O | 369 | A | 37 | 3 | 78 | 80 | 387.78 | 72.83 | 0.5117683 | -16.97 | **-16.29** | 0.20 |

[a–f] Footnotes as for Table 2.

**Supplementary Table 6.** Nd isotope results for Site U1403

| Sample ID[a] | Lab[b] | Site | Hole | Core | Sec. | from (cm) | to (cm) | Depth (rCCSF) | Age (Ma)[c] | $^{143}Nd/^{144}Nd$ | $\varepsilon_{Nd(0)}$[d] | $\boldsymbol{\varepsilon_{Nd(t)}}$[e] | 2σ[f] |
|---|---|---|---|---|---|---|---|---|---|---|---|---|---|
| *leachates* | | | | | | | | | | | | | |
| 34 | K | 1403 | A | 22 | 1 | 118 | 120 | 211.17 | 58.38 | 0.51220316 | -8.48 | **-7.94** | 0.28 |
| 41 | K | 1403 | B | 25 | 3 | 32 | 34 | 214.92 | 59.27 | 0.51219441 | -8.65 | **-8.1** | 0.28 |
| 39 | K | 1403 | B | 25 | 5 | 110 | 112 | 218.7 | 60.16 | 0.5121726 | -9.08 | **-8.52** | 0.28 |
| 26 | K | 1403 | A | 23 | 4 | 130 | 132 | 222.04 | 60.95 | 0.51211773 | -10.15 | **-9.58** | 0.28 |
| 36 | K | 1403 | B | 26 | 3 | 17 | 19 | 225.83 | 61.85 | 0.51206354 | -11.21 | **-10.63** | 0.28 |
| 38 | K | 1403 | A | 25 | 1 | 88 | 90 | 233.61 | 63.50 | 0.51207215 | -11.04 | **-10.45** | 0.28 |
| 61 | K | 1403 | A | 25 | 3 | 14 | 16 | 235.87 | 63.96 | 0.51214574 | -9.6 | **-9.01** | 0.41 |
| 45 | K | 1403 | A | 25 | 6 | 3 | 5 | 240.26 | 64.84 | 0.51212113 | -10.08 | **-9.48** | 0.28 |
| 29 | K | 1403 | A | 26 | 1 | 18 | 20 | 241.77 | 65.00 | 0.51208917 | -10.71 | **-10.1** | 0.41 |
| 46 | K | 1403 | A | 26 | 4 | 8 | 10 | 246.17 | 65.49 | 0.51209801 | -10.53 | **-9.93** | 0.28 |
| 46b | O | 1403 | A | 26 | 4 | 8 | 10 | 246.17 | 65.49 | 0.51209984 | -10.5 | **-9.89** | 0.20 |
| 64 | K | 1403 | A | 26 | 4 | 68 | 70 | 246.77 | 65.71 | 0.51209701 | -10.55 | **-9.95** | 0.41 |
| 47 | K | 1403 | B | 28 | 1 | 15 | 16.5 | 247.16 | 65.85 | 0.51211903 | -10.12 | **-9.51** | 0.28 |
| 63 | K | 1403 | B | 28 | 1 | 30 | 32 | 247.31 | 65.90 | 0.51210475 | -10.4 | **-9.79** | 0.41 |
| 48 | K | 1403 | B | 28 | 1 | 45 | 46.5 | 247.46 | 65.96 | 0.51214753 | -9.57 | **-8.96** | 0.28 |
| 48b | O | 1403 | B | 28 | 1 | 45 | 46.5 | 247.46 | 65.96 | 0.5121445 | -9.63 | **-9.02** | 0.20 |
| 68 | K | 1403 | B | 28 | 1 | 60 | 62 | 247.601 | 66.01 | 0.51210539 | -10.39 | **-9.78** | 0.41 |
| 49 | K | 1403 | B | 28 | 1 | 75 | 76.5 | 247.76 | 66.05 | 0.51208094 | -10.87 | **-10.26** | 0.28 |
| 30 | O | 1403 | B | 28 | 1 | 95 | 96.5 | 247.96 | 66.07 | 0.51214083 | -9.7 | **-9.09** | 0.13 |
| 60 | K | 1403 | B | 28 | 1 | 115 | 117 | 248.16 | 66.09 | 0.51214344 | -9.65 | **-9.04** | 0.41 |
| 65 | K | 1403 | B | 28 | 2 | 6 | 8 | 248.57 | 66.13 | 0.51211151 | -10.27 | **-9.66** | 0.41 |
| 50 | K | 1403 | B | 28 | 2 | 46.5 | 48 | 248.97 | 66.17 | 0.51216274 | -9.27 | **-8.66** | 0.28 |
| 50b | O | 1403 | B | 28 | 2 | 46.5 | 48 | 248.97 | 66.17 | 0.51214194 | -9.68 | **-9.06** | 0.20 |
| 35 | K | 1403 | A | 27 | 2 | 30 | 32 | 253.95 | 66.68 | 0.51211607 | -10.18 | **-9.56** | 0.28 |
| 32 | K | 1403 | A | 27 | 3 | 100 | 102 | 256.15 | 66.90 | 0.51210387 | -10.42 | **-9.8** | 0.41 |
| 51 | K | 1403 | A | 27 | 5 | 90 | 92 | 259.05 | 67.18 | 0.51208445 | -10.8 | **-10.18** | 0.28 |
| 37 | K | 1403 | B | 29 | 3 | 130 | 132 | 263.28 | 67.58 | 0.51209507 | -10.59 | **-9.97** | 0.28 |
| 52 | K | 1403 | A | 28 | 4 | 30 | 32 | 267.75 | 67.92 | 0.51212524 | -10 | **-9.37** | 0.28 |
| 52b | O | 1403 | A | 28 | 4 | 30 | 32 | 267.75 | 67.92 | 0.51213415 | -9.83 | **-9.2** | 0.20 |
| 27 | K | 1403 | B | 30 | 2 | 60 | 62 | 271.25 | 68.33 | 0.51213766 | -9.76 | **-9.13** | 0.28 |
| 67 | K | 1403 | B | 31 | 1 | 1 | 3 | 279.83 | 69.83 | 0.51213027 | -9.9 | **-9.26** | 0.41 |
| 42 | K | 1403 | B | 31 | 3 | 100 | 102 | 283.82 | 70.63 | 0.51217083 | -9.11 | **-8.46** | 0.28 |
| 44 | K | 1403 | B | 32 | 1 | 3 | 5 | 290.52 | 71.97 | 0.51220812 | -8.39 | **-7.72** | 0.28 |
| 44b | O | 1403 | B | 32 | 1 | 3 | 5 | 290.52 | 71.97 | 0.51222027 | -8.15 | **-7.48** | 0.20 |
| 62 | K | 1403 | B | 32 | 3 | 30 | 32 | 293.79 | 72.63 | 0.51214667 | -9.58 | **-8.91** | 0.41 |
| 40 | K | 1403 | B | 32 | 5 | 40 | 42 | 296.59 | 73.19 | 0.51220583 | -8.43 | **-7.75** | 0.28 |
| *detrital fractions* | | | | | | | | | | | | | |
| 54TD | K | 1403 | B | 25 | 3 | 32 | 34 | 214.92 | 59.27 | 0.51211421 | -10.22 | **-9.67** | 0.18 |
| 53TD | K | 1403 | A | 25 | 1 | 88 | 90 | 233.61 | 63.50 | 0.51197308 | -12.97 | **-12.38** | 0.18 |
| 123TD | K | 1403 | A | 26 | 1 | 18 | 20 | 241.77 | 65.00 | 0.5120373 | -11.72 | **-11.12** | 0.18 |
| 56TD | K | 1403 | A | 26 | 4 | 8 | 10 | 246.17 | 65.49 | 0.51200563 | -12.34 | **-11.73** | 0.18 |
| 124TD | K | 1403 | A | 28 | 4 | 30 | 32 | 267.75 | 67.92 | 0.51205968 | -11.28 | **-10.65** | 0.18 |
| 125TD | K | 1403 | B | 31 | 1 | 1 | 3 | 279.83 | 69.83 | 0.51204884 | -11.49 | **-10.85** | 0.18 |
| 55TD | K | 1403 | B | 32 | 1 | 3 | 5 | 290.52 | 71.97 | 0.51211144 | -10.27 | **-9.61** | 0.18 |

[a–f] Footnotes as for Table 2.

**Supplementary Table 7.** Tie-points used for the construction of age-models

| Site 516<br>Stratigraphic event[a] | Depth<br>(mcd) | Age<br>(Ma) |
|---|---|---|
| base C24n3n | 888.10 | 53.983 |
| base NP10 | 898.50 | 55.860 |
| base NP9 | 913.80 | 57.210 |
| base C25N | 916.72 | 57.656 |
| base NP8 | 920.10 | 58.700 |
| base NP7 | 929.10 | 58.970 |
| base NP6 | 936.60 | 59.540 |
| base NP5 | 943.30 | 61.510 |
| base NP4 | 946.10 | 63.250 |
| base NP3 | 962.10 | 64.810 |
| base NP2 | 963.10 | 65.470 |
| K/Pg boundary | 964.11 | 66.040 |
| base C29r | 971.42 | 66.398 |
| base C31n | 1011.15 | 69.269 |
| base C31r | 1032.03 | 71.449 |
| base C32r | 1071.88 | 74.309 |

| Site 525<br>Stratigraphic event[a] | Depth<br>(rmcd) | Age<br>(Ma) |
|---|---|---|
| base NP10 | 371.30 | 55.860 |
| base NP9 | 409.40 | 57.210 |
| base NP8 | 415.90 | 58.700 |
| base NP7 | 416.70 | 58.970 |
| base NP6 | 423.50 | 59.540 |
| base NP5 | 433.00 | 61.510 |
| base NP4 | 442.38 | 63.250 |
| base NP3 | 446.80 | 64.810 |
| base NP2 | 449.30 | 65.470 |
| K/Pg boundary | 451.71 | 66.040 |
| base C29r | 456.69 | 66.389 |
| base C30n | 475.15 | 68.196 |
| base C30r | 476.40 | 68.369 |
| base C31n | 489.35 | 69.269 |
| base C31r | 519.95 | 71.449 |
| base C32n1n | 522.61 | 71.689 |
| base C32n1r | 526.24 | 71.939 |
| C32n2n, estimate<br>(base C32n2n, age 73.649, below 565 m) | 565.00 | 73.600 |

| Site 1267<br>Stratigraphic event[a] | Depth<br>(rmcd) | Age<br>(Ma) |
|---|---|---|
| astrochronology | 232.05 | 56.042 |
| astrochronology | 232.63 | 56.105 |
| astrochronology | 232.85 | 56.126 |
| astrochronology | 233.10 | 56.147 |
| astrochronology | 233.65 | 56.189 |
| astrochronology | 233.88 | 56.21 |
| astrochronology | 235.40 | 56.315 |
| astrochronology | 236.95 | 56.399 |
| astrochronology | 237.43 | 56.42 |

| | | |
|---|---|---|
| astrochronology | 237.80 | 56.441 |
| astrochronology | 239.20 | 56.504 |
| astrochronology | 239.55 | 56.525 |
| astrochronology | 240.30 | 56.567 |
| astrochronology | 240.68 | 56.588 |
| astrochronology | 241.05 | 56.609 |
| astrochronology | 241.43 | 56.630 |
| astrochronology | 242.33 | 56.693 |
| astrochronology | 243.05 | 56.735 |
| astrochronology | 243.43 | 56.756 |
| astrochronology | 244.08 | 56.819 |
| astrochronology | 245.08 | 56.882 |
| astrochronology | 245.40 | 56.903 |
| astrochronology | 245.75 | 56.924 |
| astrochronology | 246.85 | 56.987 |
| astrochronology | 247.25 | 57.008 |
| astrochronology | 247.53 | 57.029 |
| astrochronology | 248.40 | 57.113 |
| astrochronology | 249.13 | 57.176 |
| astrochronology | 249.53 | 57.197 |
| astrochronology | 249.83 | 57.218 |
| astrochronology | 251.43 | 57.302 |
| astrochronology | 251.70 | 57.323 |
| astrochronology | 253.46 | 57.386 |
| astrochronology | 253.74 | 57.407 |
| astrochronology | 256.14 | 57.575 |
| astrochronology | 256.49 | 57.596 |
| astrochronology | 257.61 | 57.659 |
| astrochronology | 257.99 | 57.680 |
| astrochronology | 258.34 | 57.701 |
| astrochronology | 259.11 | 57.743 |
| astrochronology | 259.43 | 57.764 |
| astrochronology | 259.80 | 57.785 |
| astrochronology | 260.13 | 57.806 |
| astrochronology | 260.45 | 57.827 |
| astrochronology | 261.35 | 57.890 |
| astrochronology | 261.61 | 57.911 |
| astrochronology | 263.43 | 58.016 |
| astrochronology | 263.88 | 58.037 |
| astrochronology | 264.33 | 58.058 |
| astrochronology | 265.50 | 58.121 |
| astrochronology | 265.83 | 58.142 |
| astrochronology | 266.15 | 58.163 |
| astrochronology | 266.45 | 58.184 |
| astrochronology | 266.75 | 58.205 |
| base C25r | 275.48 | 58.959 |
| base C26n | 277.40 | 59.237 |
| base C26r | 299.90 | 62.221 |
| base C27n | 301.65 | 62.517 |
| base C27r | 307.08 | 63.494 |
| base C28n | 312.53 | 64.667 |
| base C28r | 313.75 | 64.958 |
| base C29n | 317.92 | 65.688 |
| K/Pg boundary | 320.25 | 66.040 |

| | | |
|---|---|---|
| base C29r | 326.88 | 66.389 |
| extrapolated | 366.30 | 69.076 |

| Site 369<br>Stratigraphic event[a] | Depth<br>(mbsf) | Age<br>(Ma) |
|---|---|---|
| extrapolated | 372.00 | 66.916 |
| base C31n | 376.00 | 69.269 |
| base *Micula mura* zone | 377.00 | 69.000 |
| average of two events above | 376.50 | 69.135 |
| base C31r | 379.00 | 71.449 |
| top *Globotruncana gansseri* zone | 384.00 | 71.750 |
| average of two events above | 381.50 | 71.600 |
| top *Eiffellithus eximius* zone | 412.00 | 75.950 |
| Top *Globotruncana elevata* zone | 412.00 | 79.200 |
| average of two events above | 412.00 | 77.575 |

| Site U1403<br>Stratigraphic event[a] | Depth<br>(rCCSF) | Age<br>(Ma) |
|---|---|---|
| base NP 10, FO *Rhomboaster bramlettei* | 195.75 | 55.860 |
| P-E boundary, FO *Rhomboaster* spp. | 200.98 | 55.960 |
| base *Lithoptychius* spp., 1st radiation fasc. | 227.00 | 62.200 |
| base NP3, FO *Chiasmolithus danicus* | 239.98 | 64.810 |
| base NP2, FO *Cruciplacolithus intermedius* | 246.12 | 65.470 |
| K/Pg boundary | 247.69 | 66.040 |
| astrochronology | 251.04 | 66.374 |
| astrochronology | 254.96 | 66.782 |
| astrochronology | 259.21 | 67.192 |
| astrochronology | 263.37 | 67.585 |
| astrochronology | 268.68 | 67.986 |
| astrochronology | 271.64 | 68.383 |
| astrochronology | 274.64 | 68.787 |
| astrochronology | 275.00 | 68.860 |
| extrapolated | 300.00 | 73.870 |

a) "C" followed by a number: magnetochron, "NP" followed by a number: nannoplankton zone[14], LO: Last Occurrence, FO: First occurrence. In case of contradicting ages for stratigraphic events for Site 369, average depths and ages were used as tie-points.

**References**


1. Barker, P.F., Carlson, R.L. & Johnson, D.A. Init. Rep. Deep Sea Drill. Proj. 72 (1983).
2. Berggren, W.A. *et al*. Magnetobiostratigraphy of Deep Sea Drilling Project Leg 72, Sites 515-518, Rio Grande Rise (South Atlantic). In: Barker, P.F. *et al*., Init. Rep. Deep Sea Drill. Proj. 72, 938–948 (1983).
3. Gradstein, F., Ogg, J., Schmitz, M., Ogg, G. (Eds.) *The Geologic Time Scale 2012*. (Elsevier, 2012).
4. Moore, T.C., Rabinowitz, P.D. *et al*., Init. Rep. Deep Sea Drill. Proj. 74 (1984).
5. Li, L. & Keller, G. Abrupt deep-sea warming at the end of the Cretaceous. *Geology* **26**, 995–998 (1998).
6. Chave, A.D. Lower Paleocene-Upper Cretaceous magnetostratigraphy, Sites 525, 527, 528, and 529, Deep Sea Drilling Project Leg 74, in: Moore, T.C. *et al*., Init. Rep. Deep Sea Drill. Proj. 74, 525–531 (1984).
7. Via, R. K. & Thomas, D. J. Evolution of Atlantic thermohaline circulation: Early Oligocene onset of deep-water production in the North Atlantic. *Geology* **34,** 441–444 (2006).
8. Zachos, J.C. *et al*. Leg 208 summary. Proceed. Ocean Drill. Progr., Init. Rep. 208, 1–112 (2004).
9. Westerhold, T. *et al*. On the duration of magnetochrons C24r and C25n and the timing of early Eocene global warming events: Implications from the Ocean Drilling Program Leg 208 Walvis Ridge depth transect. *Paleoceanography* **22**, PA2201 (2007).
10. Lancelot, Y., Seibold, E. et al. Init. Rep. Deep Sea Drill. Proj. 41 (1978).
11. Expedition 342 Scientists, IODP Prel. Rept. 342 (2012)
12. Norris, R.D., Wilson, P.A., Blum, P., & Expedition 342 Scientists Site 1403. Proceed. Integrat. Ocean Drill. Progr. 342 (2014).
13. Batenburg, S.J. et al. Late Maastrichtian carbon isotope stratigraphy and cyclostratigraphy of the Newfoundland Margin (Site U1403, IODP Leg 342). Newsl. Stratigr. 10.1127 (2017).
14. Martini, E., Standard Palaeogene calcareous nannoplankton zonation. *Nature* **226**, 560–561 (1970).
